# Supplementary material for: The causal relationship between the human gut microbiota and pyogenic arthritis: a Mendelian randomization study
Source: Front Cell Infect Microbiol. 2024 Nov 26;14:1452480. doi: 10.3389/fcimb.2024.1452480 (PMC11629706; doi:10.3389/fcimb.2024.1452480)
Supplement: Supplementary file 1 [file DataSheet1.zip › mendelian test/ebi-a-GCST90027476.csv_leave_one_out.pdf]

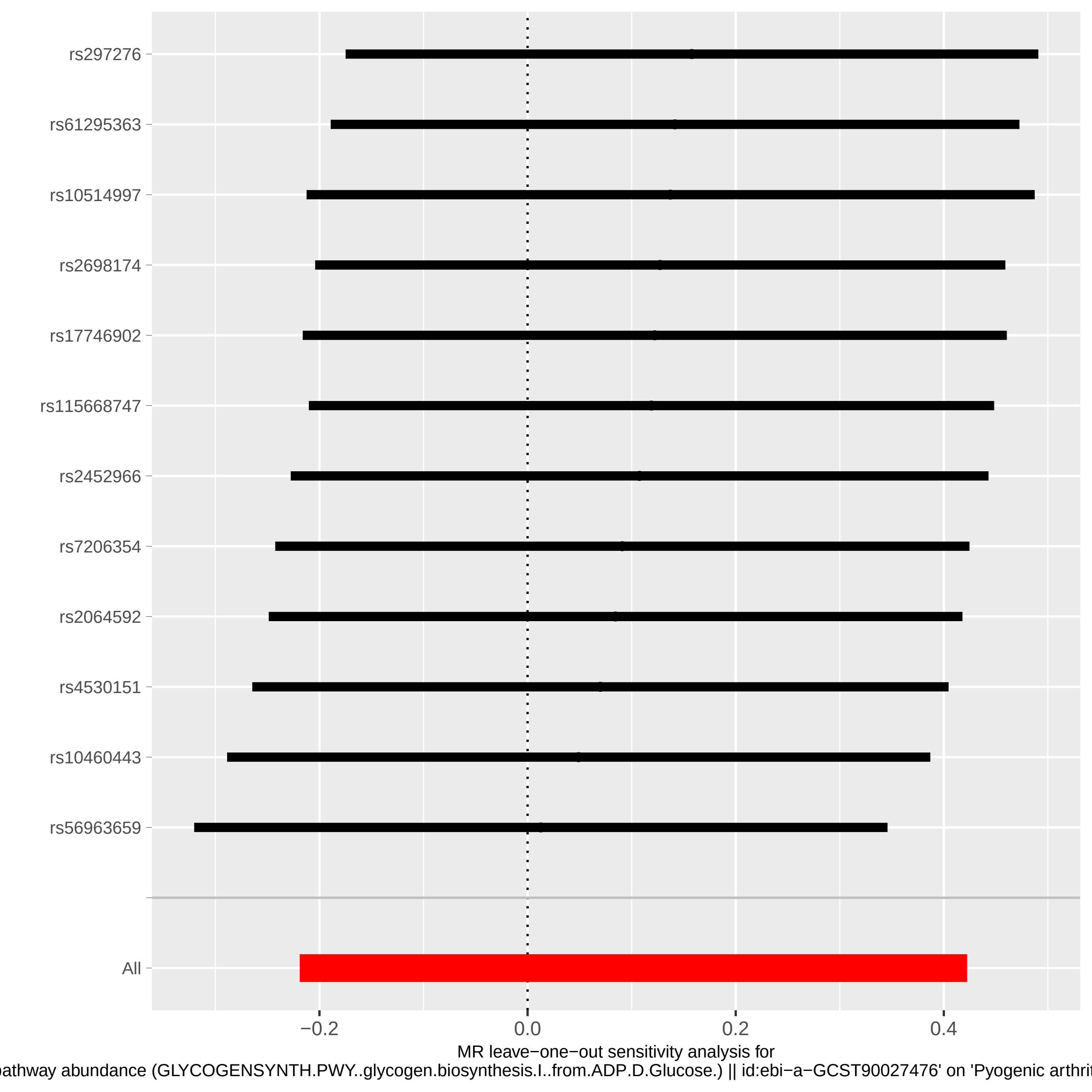
rs297276
rs61295363
rs10514997
rs2698174
rs17746902
rs115668747
rs2452966
rs7206354
rs2064592
rs4530151
rs10460443
rs56963659
All
−0.2
0.0
0.2
0.4
MR leave−one−out sensitivity analysis for
pathway abundance (GLYCOGENSYNTH.PWY..glycogen.biosynthesis.I..from.ADP.D.Glucose.) || id:ebi−a−GCST90027476' on 'Pyogenic arthrit
